# The response of litter decomposition to extreme drought modified by plant species, plant part, and soil depth in a temperate grassland

**DOI:** 10.1002/ece3.9652

**Published:** 2022-12-21

**Authors:** Anikó Seres, György Kröel‐Dulay, Judit Szakálas, Péter István Nagy, Gergely Boros, Gábor Ónodi, Miklós Kertész, Katalin Szitár, Andrea Mojzes

**Affiliations:** ^1^ Department of Zoology and Ecology, Institute for Wildlife Management and Nature Conservation Szent István Campus, Hungarian University of Agriculture and Life Sciences Gödöllő Hungary; ^2^ Centre for Ecological Research Institute of Ecology and Botany Vácrátót Hungary; ^3^ ‘Lendület’ Landscape and Conservation Ecology Group, Centre for Ecological Research Institute of Ecology and Botany Vácrátót Hungary

**Keywords:** climate change, dominance shift, litter quality, rooting depth, root‐to‐shoot ratio

## Abstract

Plant litter decomposition is a key ecosystem process in carbon and nutrient cycling, and is heavily affected by changing climate. While the direct effects of drought on decomposition are widely studied, in order to better predict the overall drought effect, indirect effects associated with various drought‐induced changes in ecosystems should also be quantified. We studied the effect of an extreme (5‐month) experimental drought on decomposition, and if this effect varies with two dominant perennial grasses, plant parts (leaves vs. roots), and soil depths (0–5 cm vs. 10–15 cm) in a semi‐arid temperate grassland. After 12 months, the average litter mass loss was 43.5% in the control plots, while only 25.7% in the drought plots. Overall, mass loss was greater for leaves (44.3%) compared to roots (24.9%), and for *Festuca vaginata* (38.6%) compared to *Stipa borysthenica* (30.5%). This variation was consistent with the observed differences in nitrogen and lignin content between plant parts and species. Mass loss was greater for deep soil (42.8%) than for shallow soil (26.4%). Collectively, these differences in decomposition between the two species, plant parts, and soil depths were similar in magnitude to direct drought effect. Drought induces multiple changes in ecosystems, and our results highlight that these changes may in turn modify decomposition. We conclude that for a reliable estimate of decomposition rates in an altered climate, not only direct but also indirect climatic effects should be considered, such as those arising from changing species dominance, root‐to‐shoot ratio, and rooting depth.

## Graphical abstract

Our field experiment showed that a 5‐month extreme drought substantially decreased the decomposition of both leaf and root litter of two dominant perennial grass species in a semi‐arid temperate grassland. In addition, we found differences in decomposition rate between the two species, plant parts (leaves vs. roots), and soil depths (0–5 cm vs. 10–15 cm) in a magnitude similar to the direct drought effect. We conclude that for a reliable estimate of decomposition rates in an altered climate, not only direct but also indirect climatic effects should be considered, such as those arising from changing species dominance, root‐to‐shoot ratio, and rooting depth.

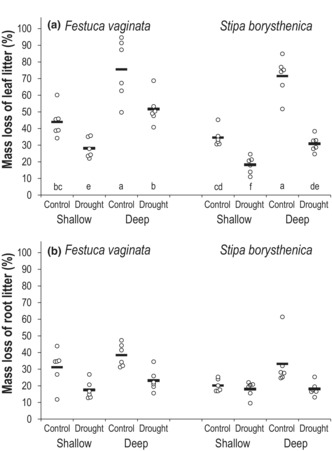

## 1 INTRODUCTION

Plant litter decomposition is a key ecological process that plays a major role in carbon and nutrient cycling, thus regulating soil carbon sequestration and soil nutrient availability (Chapin III et al., 2011). Litter decomposition rate is generally controlled by three factors: climate (mainly temperature and precipitation), litter quality (the physico‐chemical traits of the litter), and the composition and abundance of decomposer organisms, although the relative importance of these factors may vary with spatial scales and ecosystem types (Aerts, 1997; Chapin III et al., 2011; González & Seastedt, 2001; Zhang et al., 2008).

Climate change is projected to increase extreme drought risk in many areas of the world, such as western North America, southern Africa, the Amazon, and Europe, by the end of the 21st century, including even regions where wetter average conditions are predicted (e.g. East Africa; Cook et al., 2020). Extreme drought can substantially affect ecosystem carbon pools and fluxes, and the mechanisms of these responses may include drought‐induced changes in decomposition (Frank et al., 2015). However, due to the complexity of these changes (i.e., direct and indirect, immediate and lagged effects), the impacts of extreme drought on ecosystem carbon cycling are not fully understood. Grasslands (including savannas) store a large amount of soil carbon (517 Pg C), about 22% of the total terrestrial soil organic carbon storage of the world (estimated for the top 3 m of soil; Jobbágy & Jackson, 2000). Therefore, there is a need to understand how litter decomposition in grasslands responds to changing climate, with particular emphasis on the effects of extreme drought.

In arid and semi‐arid ecosystems, water availability is often the dominant factor that influences litter decomposition (Jacobson & Jacobson, 1998; Strojan et al., 1987; Zhang et al., 2016). Synthesis works of studies investigating the effect of changes in precipitation or soil moisture conditions on decomposition rate showed that in temperate or (semi‐)arid regions, decomposition usually decreased in response to drought (Walter, 2018; Wu et al., 2020). This was also found when focusing on grasslands, as in most studies conducted in temperate grasslands or experimental grassland communities, drought reduced litter decomposition (Bernard et al., 2019; Chomel et al., 2019; Mariotte et al., 2015; Sanaullah et al., 2012; Vogel et al., 2013; Walter et al., 2013; Wang et al., 2015; Yahdjian et al., 2006). However, several studies reported that drought had no effect (Bernard et al., 2019; Vogel et al., 2013), or even increased decomposition (Haugwitz et al., 2016; Kreyling et al., 2008). Furthermore, Reed et al. (2009) found that a decrease in decomposition rate during the drought period switched to an increase one year after the cessation of the treatment. Such high variation suggests that the effect of drought on decomposition is more complex than simply the moisture limitation of soil microbial and faunal activity.

The diverse response of decomposition to drought reported in individual grassland studies may partly be related to the fact that temperature and moisture availability may regulate decomposition not only directly through affecting the biological activity (growth, survival and/or mobility) of decomposer organisms (Cruz‐Paredes et al., 2021; Manzoni et al., 2012), but also indirectly via various parallel climate‐induced changes in ecosystems, such as changes in litter chemistry (Aerts, 1997; Zhang et al., 2008). Altered climatic conditions can change litter quality within species via modification of plant physiological processes, including changes in the quantity, composition, and localization of compounds in plant tissues, which may enhance or hinder the decomposition of their litter (Suseela & Tharayil, 2018). Changes in climate may also alter litter quality by inducing a shift in plant species or functional group abundances in the community. In a subalpine dry meadow, experimental warming increased the aboveground biomass of the shrub species, *Artemisia tridentata*, but decreased the abundance of forbs (Harte & Shaw, 1995), and *A. tridentata* decomposed slower than forbs primarily due to its higher initial lignin: nitrogen ratio compared to forbs (Shaw & Harte, 2001).

Factors by which altered climatic conditions can indirectly affect plant litter decomposition may also include changes in root‐to‐shoot ratio and rooting depth. Just as litter quality, these factors can also change at species and community levels, due to altered biomass allocation within a species and shift in species abundances, respectively. An increase in root‐to‐shoot ratio within a species is a well‐documented response to drought, which allows plants to enhance water uptake from drier soils (Fernández & Reynolds, 2000; Skinner & Comas, 2010; Wang et al., 2016). As an example of changes at a community level, experimental drought in a mesic alpine grassland increased the relative abundance of deep‐rooted grasses, and decreased that of shallow‐rooted sedges and forbs, which contributed to a shift toward higher belowground net primary productivity compared to control, particularly in the deeper soil layer (Liu et al., 2018). Drought‐induced shift of biomass allocation to belowground was also evidenced by a recent meta‐analysis of drought experiments in grasslands due to the decrease of aboveground biomass in response to precipitation reduction (Zhang & Xi, 2021). Altered root‐to‐shoot ratio in response to drought affects decomposition often, but not always, via changes in litter quality. In temperate grasslands, belowground or root litter generally decomposes slower than aboveground or leaf litter mostly due to the lower nitrogen content and/or higher lignin content of roots compared to leaves or shoots (Semmartin et al., 2004; Vivanco & Austin, 2006; Wang et al., 2015). However, environmental factors, such as higher and less variable moisture content, and a greater area of contact with the soil belowground, may override the effects of litter quality, resulting in faster decomposition of roots compared to shoots (Tahir et al., 2016). Although many studies have investigated belowground litter decomposition at multiple soil depths in grasslands, there is no general consensus on the effect of depth on decomposition. Some studies reported that belowground decomposition rate decreased gradually with depth (Gill & Burke, 2002; Von Haden & Dornbush, 2019), while others found faster decomposition in the deeper soil layer compared to the shallow layer (Castanha et al., 2018; Garcia‐Pausas et al., 2012). Collectively, these previous studies suggest that for a better understanding of the drought response of litter decomposition in temperate grasslands, differences in decomposition rate between different plant species, plant parts, and soil depths should also be investigated.

The objective of this study was to assess the effects of an extreme (5‐month) experimental drought on the leaf and root decomposition of two dominant perennial grass species (*Festuca vaginata* and *Stipa borysthenica*) at two soil depths (0–5 cm and 10–15 cm, hereafter referred to as shallow and deep soil layer, respectively) in a semi‐arid sand grassland in Hungary. We also examined the initial litter quality of leaves and roots of the two species. We hypothesized that (H_1_) drought decreases decomposition, (H_2_) the species having poorer initial litter quality (lower nitrogen and higher lignin content) shows a lower decomposition rate, (H_3_) leaves decompose more quickly than roots, and (H_4_) decomposition is faster in the deep soil layer than in the shallow layer. The deep soil layer may provide more favorable temperature and moisture conditions for biological activity compared to the shallow layer.

## 2 MATERIALS AND METHODS

### 2.1 Study site

The study site (46.870° N, 19.422° E; 108 m above sea level) is located in the Danube–Tisza Interfluve (Central Hungary), near the village Fülöpháza, in the Kiskunság National Park. The climate of the region is temperate continental with sub‐Mediterranean influences. Mean annual temperature is 10.8°C, and mean annual precipitation is 570.9 mm (2001–2013) at Fülöpháza, based on a standard meteorological station located next to the study site. The soil is nutrient‐poor, coarse‐textured calcareous sandy soil with >95% sand content, ca. 11% CaCO_3_, and <1% humus content in the upper 30‐cm layer, and low water‐holding capacity (Kovács‐Láng et al., 2000). This extreme water and nutrient regime of the soil amplifies mid‐summer droughts typical in July and August. Recent climate models projected a decrease in precipitation amount and in the frequency of rainy days with a simultaneous increase in the maximum number of consecutive dry days in summer for Hungary by the end of the 21st century (Torma et al., 2020). Furthermore, in a multi‐indicator sensitivity analysis, the sand dune region of the Kiskunság National Park was found to be one of the most sensitive areas to drought (Ladányi et al., 2015). The studied vegetation is a semi‐arid sand grassland, characterized by the dominance of two perennial bunchgrasses, *Festuca vaginata* Waldst. and Kit. ex Willd. and *Stipa borysthenica* Klokov ex Prokudin.

### 2.2 Rainfall manipulation experiment

In 2014, we set up a field experiment, where experimental units were 3 m × 3 m plots with a 50‐cm buffer strip along the inner margin of each side of the plot (i.e., the effective sampling area was 2 m × 2 m). Plots were arranged in six blocks, where each block contained one control and one drought plot (six replicates per treatment, 12 plots in total). Control plots received ambient rainfall, while in the drought plots, we conducted an extreme drought treatment from 24 April to 18 September 2014 by excluding rain using fixed, transparent polyethylene roofs. Side curtains were used to prevent rain from entering the drought plots from the side. This 5‐month drought treatment exceeded the length of historically observed natural droughts in the study area (most frequently 2 months in July–August or August–September; Kun, 2001).

Soil temperature was measured at 10 cm depth of the soil (i.e., probes were inserted horizontally into the soil), and volumetric soil water content (%) was recorded at 0–30 cm depth (i.e., averaged over the soil profile) in each plot by permanent temperature and moisture sensors (Jumo RTD Pt‐100 and Campbell CS616, respectively) connected to a data logger. The amount of precipitation was measured with rain gauges (Davis DS7852) at 30‐cm height. The canopy cover of *F. vaginata* and *S. borysthenica* was visually estimated in four 1 m × 1 m quadrats in each plot in April and September 2014 (i.e., before and after the drought treatment). Data of the four quadrats were averaged for each plot.

### 2.3 Background conditions for decomposition: Precipitation, microclimate, and the abundance of dominant grasses

In 2014, annual precipitation was 43% higher than the average (570.9 mm) of the previous 13 years (2001–2013; Table 1). The 5‐month experimental drought treatment excluded 64% of ambient annual precipitation (523.5 mm). During the treatment period on average, rain exclusion decreased soil water content to 3.3% compared to control (5.5%), while soil temperature was 1.5°C higher in the drought plots than in the control plots (Table 1, Figure S1).

**TABLE 1:** Precipitation (yearly total), daily average volumetric soil water content, and daily average soil temperature during the drought treatment period (between 24 April and 18 September 2014) and the percentage cover of the two dominant perennial grasses, *Festuca vaginata* and *Stipa borysthenica* in the experimental plots in April and September 2014 (i.e., before and after the drought treatment).

	Control	Drought
Precipitation (mm)	817.3	293.8
Soil water content (%) at 0–30 cm	5.5 ± 0.1	3.3 ± 0.1
Soil temperature (°C) at 10 cm	23.4 ± 0.3	24.9 ± 0.3
Cover (%) of *F. vaginata* in April	4.5 ± 1.1	3.7 ± 0.8
Cover (%) of *S. borysthenica* in April	5.0 ± 1.1	4.0 ± 1.0
Cover (%) of *F. vaginata* in September	6.8 ± 1.4	0.2 ± 0.1
Cover (%) of *S. borysthenica* in September	7.1 ± 1.0	1.0 ± 0.2

*Note*: For soil water content, temperature, and plant cover, values are means ± SE (*n* = 6).

In April 2014, prior to the start of the drought treatment, the percentage cover of *F. vaginata* and *S. borysthenica* was similar in the control and treatment plots. Rain exclusion caused dieback of both species, but the decline was greater for *F. vaginata* than for *S. borysthenica* (Table 1). This resulted in five times higher abundance of *S. borysthenica* compared to that of *F. vaginata* in the drought plots at the end of the rain exclusion (September 2014). By contrast, in the control plots, these two grass species remained co‐dominant in September, and their cover even increased relative to April levels (Table 1).

### 2.4 Field sampling and laboratory analyses

We assessed litter decomposition using the minicontainer (MC) system, which is a modification of the litterbag technique, the most commonly used technique for studying decomposition in the field (Eisenbeis et al., 1999). Like the litterbag method, the minicontainer system can be used in various terrestrial ecosystems, including grasslands (Knacker et al., 2003; Kreyling et al., 2008; Mariotte et al., 2015). The MC system we used, contained a 25‐cm long PVC bar with six holes, which carried the MCs (Figure S2). The holes are 16 mm in diameter with a distance of 21 mm between the centres of the holes. Each MC consists of a central cylinder (height 16 mm, diameter 11 mm) and two end rings. Both sides of the cylinder are closed with plastic gauze discs fixed by the end rings (Eisenbeis et al., 1999). The advantage of the method is that the PVC bars can be inserted into the soil profile in close contact with the surrounding soil, with minimum disturbance and high stability, thus providing an opportunity to examine decay rates in different soil depths. Although this method requires plant material to be cut into smaller pieces due to the small size of the MCs (ca. 1.5 cm^3^), which may slightly accelerate decomposition (Eisenbeis et al., 1999), we used the same materials in each plot, thus allowing within‐site comparison.

On 26 March 2014, eight PVC bars with four MCs in each (for two soil depths and two plant parts) were placed randomly to each of the 12 plots. In each plot, four bars were filled with roots and leaves of *F. vaginata*, and four ones with those of *S. borystheni*c*a*. *Festuca vaginata* and *S. borysthenica* plants were excavated outside the experimental plots in November 2013 in order to minimize disturbance within the plots, and roots were separated from leaves. Plant material was cleaned and air‐dried at room temperature, and stored in paper bags until used for the experiment. Before the experiment, plant material was cut into pieces of ca. 1 cm in length. We used the two upper (first and second) and the two bottom (fifth and sixth) holes of the 6‐hole PVC bars. The first and fifth holes of each bar were filled with leaves, and the second and sixth holes were filled with roots (Figure S2). An amount of ca. 0.15 g (exact initial mass in each MC was measured to three decimals) plant material was placed into each MC. MCs were closed with plastic gauze discs of 2‐mm mesh size, which was permeable for micro‐ and mesofauna, but excluded macrofauna; and the bars were inserted vertically into the soil. The two upper holes were placed into the 0–5 cm layer of the soil (first and second MCs at 0–2.5 cm and 2.5–5 cm depth, respectively), and the two bottom holes were placed into the 10–15 cm layer (fifth and sixth MCs at 10–12.5 cm and 12.5–15 cm depth, respectively). MCs were retrieved after 2, 4, 6, and 12 months. In total, we used 96 PVC bars with 384 MCs (two plant species × four time periods × 12 plots). At each retrieval date, two bars per plot (one with *F. vaginata* and one with *S. borysthenica*) were collected, and immediately wrapped in parafilm (Sigma) to prevent the plant material from scattering. The samples were air‐dried at room temperature for 2 weeks, and then MCs were disassembled. The roots and leaves were cleaned from soil particles, oven‐dried at 40°C to constant mass, and weighed separately. Decomposition rate was expressed as mass loss (the percentage of initial litter mass) at each retrieval date (Seres et al., 2022).

We analyzed the initial litter quality of both leaves and roots of *F. vaginata* and *S. borysthenica* collected outside the experimental plots. Carbon (C) and nitrogen (N) contents were measured in five replicates with a Carlo Erba (Fisons) NA 1500 element analyzer, and C:N ratio was calculated. Lignin, cellulose, and hemicellulose (hereafter jointly referred to as fibers) content of plants was determined in 10 replicates by using the acid‐detergent fiber method (Van Soest, 1963). The content of each chemical component was expressed as the percentage of initial litter dry mass (Seres et al., 2022).

### 2.5 Statistical analysis

**FIGURE 1:**
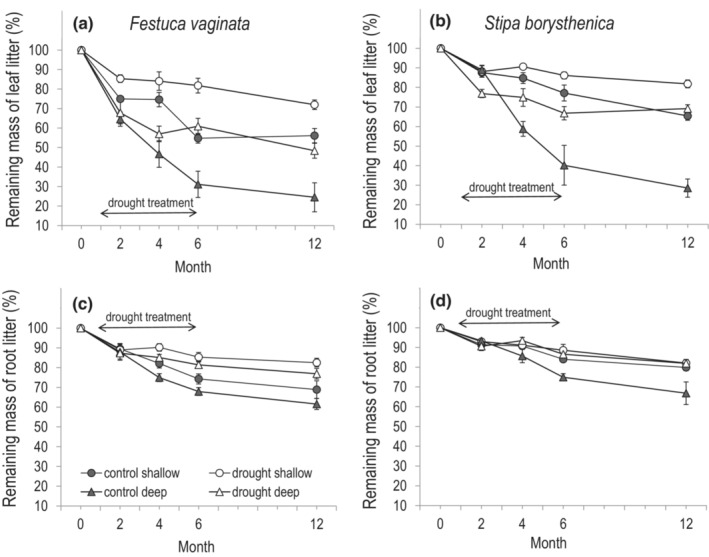
Litter decomposition dynamics of (a, c) *Festuca vaginata* and (b, d) *Stipa borysthenica* at two soil depths of the control and drought plots. Remaining mass (the percentage of initial mass) for (a, b) leaf litter and (c, d) root litter at the four retrieval dates (2, 4, 6, and 12 months) of the experiment. Shallow soil layer denotes 0–2.5 cm for leaves and 2.5–5 cm for roots. Deep soil layer denotes 10–12.5 cm for leaves and 12.5–15 cm for roots. Values are mean ± SE. Arrows indicate the timing of the drought treatment.

Each statistical analysis was performed in the R programming environment (version 4.1.1; R Core Team, 2021). The effects of explanatory variables, i.e., treatment (control vs. drought), species (*F. vaginata* or *S. borysthenica*), soil depth (shallow vs. deep soil layer), and time (2, 4, 6, or 12 months) on percentage litter mass loss were tested by applying linear mixed‐effects (LME) models with the explanatory variables and their interactions as fixed factors, and block as a random effect to deal with repeated measurements in the same plots (‘nlme’ package; Pinheiro et al., 2020). Data for leaves and roots were analyzed separately because of the slightly different soil layers (0–2.5 cm and 10–12.5 cm for leaves, and 2.5–5 cm and 12.5–15 cm for roots). As the average mass loss did not change substantially between the sixth and twelfth months for either species or plant parts (Figure 1a–d), we focused on the 12‐month results. For these data, we used separate linear models (LMs) to test the effects of treatment, species, and soil depth as explanatory variables and their interactions on percentage mass loss. In each analysis, we applied backward stepwise model selection from the full model by sequentially removing the non‐significant terms using the log‐likelihood ratio test until we arrived at the minimum adequate model (Zuur et al., 2009).

We used generalized least squares (GLS) models to assess the effects of species and plant part (leaf vs. root) as explanatory variables and their interaction on the initial litter quality (C and N contents, C:N ratio, and the contents of lignin, cellulose, and hemicellulose). With the use of GLS models (instead of LMs), we were able to account for heteroscedasticity (see below).

In each case, model residuals were checked for normality and homogeneity of variances by visual assessment of diagnostic plots. Percentage mass loss data were square root transformed, and C:N ratio data were square transformed for the subsequent LME and GLS analysis, respectively, to fulfill test assumptions. When model residuals still showed heteroscedasticity, we incorporated a ‘varIdent’ variance structure function into the model to allow for different residual spread per each level of one or more explanatory variables (‘nlme’ package; Pinheiro et al., 2020). We used the explanatory variable(s) in the variance structure that significantly improved the model based on log‐likelihood ratio test according to Zuur et al. (2009). For both litter quality and the 12‐month mass loss data, when we found significant interactions between the explanatory variables, we performed one degree of freedom Wald tests as post hoc tests by using the ‘contrast’ package (Kuhn et al., 2016). For the 12‐month mass loss data, we controlled for the false discovery rate in multiple comparisons by the method of Benjamini and Hochberg (1995).

## 3 RESULTS

### 3.1 Litter decomposition

Extreme drought generally decreased the mass loss of both leaf and root litter of the two species compared to control, although this effect varied with plant species, soil depth, and/or time (Figure 1a–d, Table S1).

**FIGURE 2:**
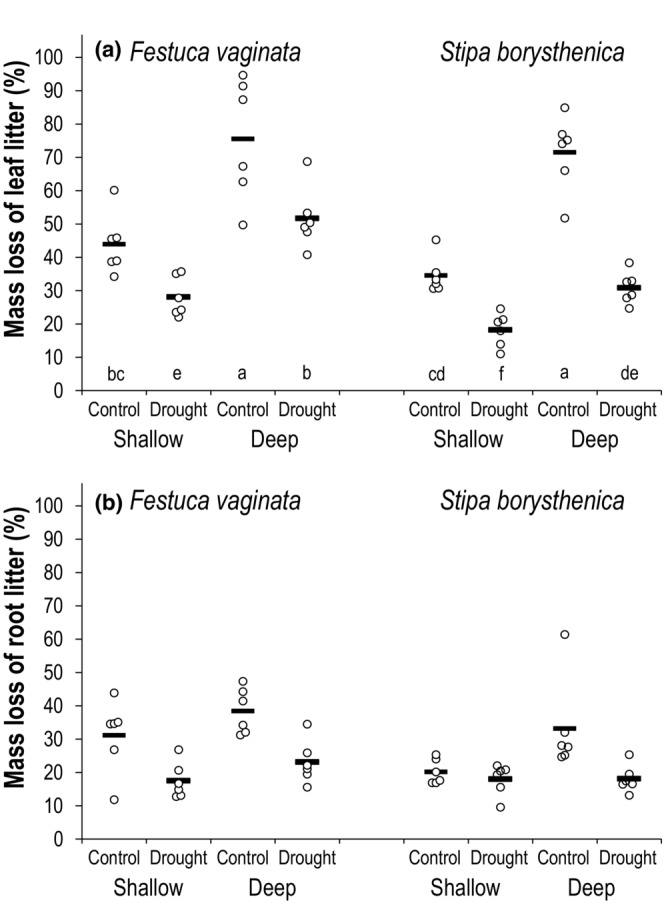
Mass loss (the percentage of initial mass) for (a) leaf litter and (b) root litter of *Festuca vaginata* and *Stipa borysthenica* at two soil depths of the control and drought plots at the end of the experiment (after 12 months). Shallow soil layer denotes 0–2.5 cm for leaves and 2.5–5 cm for roots. Deep soil layer denotes 10–12.5 cm for leaves and 12.5–15 cm for roots. Data are shown as univariate scatterplots, where circles represent individual data points (*n* = 6 per treatment). Data points are jittered along the x‐axis so that points with similar values can be visualized. Horizontal bars denote the mean values. Post hoc test was only performed for leaf litter, where we found a significant interaction between the explanatory variables in the linear model (species × treatment interaction; Tables 2 and S2). Different letters below the scatterplots indicate significant (*p* < .05) differences. Graphs were created using the template from Weissgerber et al. (2015).

After 12 months, the average mass loss was 43.5 ± 3.1% (mean ± SE) in the control plots, while only 25.7 ± 1.8% in the drought plots. Drought treatment significantly decreased root mass loss from 30.7 ± 2.3% (control) to 19.1 ± 1.1% (Tables 2 and S2, Figure 2b). For leaves, drought consistently decreased the decomposition rate of the two species in both the shallow and deep soil layers, although treatment had an effect in interaction with plant species (Tables 2 and S2, Figure 2a). Overall, mass loss was much higher for leaves (44.3 ± 3.1%) compared to roots (24.9 ± 1.5%), and tended to be greater for *F. vaginata* (38.6 ± 2.9%) than for *S. borysthenica* (30.5 ± 2.7%). For roots, the difference between the two species was independent of drought treatment and soil depth (Tables 2 and S2). For leaves, the decomposition rate of the two species differed only in the drought plots: mass loss was 39.8 ± 4.2% for *F. vaginata* and 24.5 ± 2.3% for *S. borysthenica* (Tables 2 and S2, Figure 2a). In general, mass loss was greater in the 10–15 cm layer of the soil (42.8 ± 3.2%) than in the 0–5 cm layer (26.4 ± 1.6%), and the impact of soil depth was significant for both leaves and roots (Tables 2 and S2, Figure 2a,b).

**TABLE 2:** Results of linear models (LMs) for the 12‐month data of the percentage mass loss of leaf and root litter with explanatory variables, i.e., treatment (control vs. drought), species (*Festuca vaginata* or *Stipa borysthenica*), and soil depth (shallow vs. deep soil layer) and their interactions (minimum adequate models, where nonsignificant interaction terms were removed during the model selection).

Source of variation		Leaves			Roots	
	Df	*F*	*p*	Df	*F*	*p*
Treatment	1	85.65	**<.001**	1	27.24	**<.001**
Species	1	20.76	**<.001**	1	5.46	**.024**
Depth	1	97.28	**<.001**	1	8.83	**.005**
Treatment × Species	1	4.78	**.034**			

*Note*: Data for leaves and roots were analyzed separately. Df, *F*, and *p* values denote the degrees of freedom, test statistics, and significance levels, respectively. Bold *p* values are significant at .05. For the results of full models, see Table S2.

### 3.2 Initial litter quality

For each studied chemical component, we found strong significant interactions between species and plant part (leaf vs. root), except for lignin content, where both species and plant part were significant as main effects without interaction (Tables 3 and S3).

**TABLE 3:** Results of generalized least squares (GLS) models for initial litter quality (chemical composition) including carbon (C) and nitrogen (N) contents, C:N ratio, and the contents of lignin, cellulose, and hemicellulose with explanatory variables, i.e., species (*Festuca vaginata* or *Stipa borysthenica*) and plant part (leaf vs. root), and their interaction (minimum adequate models, where the nonsignificant species × plant part interaction term was removed during the model selection for lignin).

Source of variation	Df	*χ* ^2^	*p*
Carbon (C)			
Species	1	6.96	**.008**
Plant part	1	39.56	**<.001**
Species × Plant part	1	36.26	**<.001**
Nitrogen (N)			
Species	1	281.17	**<.001**
Plant part	1	535.65	**<.001**
Species × Plant part	1	164.23	**<.001**
C:N ratio			
Species	1	291.07	**<.001**
Plant part	1	354.23	**<.001**
Species × Plant part	1	163.25	**<.001**
Lignin			
Species	1	4181.70	**<.001**
Plant part	1	15642.30	**<.001**
Cellulose			
Species	1	1.21	.272
Plant part	1	1595.00	**<.001**
Species × Plant part	1	463.45	**<.001**
Hemicellulose			
Species	1	405.60	**<.001**
Plant part	1	388.68	**<.001**
Species × Plant part	1	219.84	**<.001**

*Note*: Df, *χ* ^2^, and *p* values denote the degrees of freedom, test statistics, and significance levels, respectively. Bold *p* values are significant at .05. For the results of full models, see Table S3.

Carbon and nitrogen content was higher, while C:N ratio was lower for leaves compared to roots in both species (Table 4). *Stipa borysthenica* had lower N content and higher C:N ratio than *F. vaginata* in both leaves and roots. Lignin content was higher for roots (16.5 ± 0.5%) compared to leaves (7.4 ± 0.5%), and for *S. borysthenica* (13.9 ± 1.0%) compared to *F. vaginata* (9.9 ± 1.0%). Roots contained a higher percentage of cellulose and a lower percentage of hemicellulose than leaves for both species (Table 4). These two components differed between the two species in the opposite direction for leaves and roots. The leaves of *S. borysthenica* contained a higher percentage of hemicellulose and a lower percentage of cellulose than the leaves of *F. vaginata*, while for roots, the reverse was true: *S. borysthenica* had lower hemicellulose content and higher cellulose content than *F. vaginata* (Table 4).

**TABLE 4:** Initial quality (chemical composition) of the leaf and root litter of *Festuca vaginata* and *Stipa borysthenica*. Carbon (C) and nitrogen (N) contents, C:N ratio, and the contents of lignin, cellulose, and hemicellulose (mean ± SE) expressed as the percentage of initial litter dry mass. Sample size was five replicates for C and N contents, and C:N ratio, and 10 replicates for lignin, cellulose, and hemicellulose contents.

	*Festuca vaginata*		*Stipa borysthenica*	
	Leaf	Root	Leaf	Root
C (%)	45.2 ± 0.3^b^	44.1 ± 0.2^c^	46.7 ± 0.0^a^	40.5 ± 0.8^d^
N (%)	1.2 ± 0.0^a^	1.0 ± 0.0^c^	1.1 ± 0.0^b^	0.6 ± 0.0^d^
C:N	37.7 ± 0.6^c^	42.5 ± 0.9^b^	41.1 ± 0.6^b^	70.5 ± 1.3^a^
Lignin (%)	5.3 ± 0.0	14.4 ± 0.1	9.4 ± 0.1	18.5 ± 0.1
Cellulose (%)	32.9 ± 0.2^c^	35.9 ± 0.1^b^	29.6 ± 0.2^d^	39.6 ± 0.2^a^
Hemicellulose (%)	27.2 ± 0.3^b^	25.5 ± 0.2^c^	29.5 ± 0.5^a^	17.8 ± 0.2^d^

*Note*: Values with different superscript letters in the same row are significantly (*p* < .05) different. Post hoc tests were only performed when the species × plant part interaction was significant in the generalized least squares model (Table S3).

## 4 DISCUSSION

### 4.1 Effect of drought

The lower mass loss of leaf and root litter in the drought plots compared to the control plots for both *F. vaginata* and *S. borysthenica* is in line with our hypothesis (H_1_) that the 5‐month experimental drought decreased the litter decomposition of the two dominant perennial grass species of semi‐arid sand grasslands. These results are most likely explained by the fact that drought reduced decomposition directly by limiting the biological activity of decomposer organisms (Cruz‐Paredes et al., 2021; Manzoni et al., 2012). Although we did not measure soil biotic activity, parallel studies at the same experimental site provided evidence for this explanation (Flórián et al., 2019; Tóth et al., 2017). Firstly, similarly to our results, Tóth et al. (2017) found that the same 5‐month extreme drought treatment decreased the mass loss of standard organic matter (rooibos tea) at 3–5 cm depth under the soil surface compared to control (i.e., independent of litter type and quality). Secondly, the density of soil‐living Collembola was 90% lower in the extreme drought plots compared to the control plots (Flórián et al., 2019). As mesofauna (mostly Collembola and Acari) usually has a major effect on decomposition rates (Chapin III et al., 2011), and may be particularly important in the study area where earthworms are rare (Kovács‐Hostyánszki et al., 2013), the decreased density of Collembola may have contributed to the lower mass loss under the extreme drought treatment in our experiment. Consistently, several previous studies in grasslands showed that reduced decomposition in response to experimental drought could be explained by a decline in soil faunal and/or microbial activity (Sanaullah et al., 2012; Vogel et al., 2013; Walter et al., 2013).

### 4.2 Effect of plant species and plant part

In general, we found lower mass loss for *S. borysthenica* than for *F. vaginata*, and the litter of *S. borysthenica* had lower nitrogen content and higher lignin content than that of *F. vaginata*. These results are consistent with our hypothesis (H_2_) that the species having poorer initial litter quality (lower N and higher lignin content) shows a lower decomposition rate. In accordance with our hypothesis (H_3_) that decomposition is faster for leaf litter than for root litter, we found lower mass loss for roots compared to leaves. This difference could also be related to the variation in initial litter quality, as we found that roots contained a lower percentage of nitrogen and a higher percentage of lignin compared to leaves. Our results are consistent with previous studies that compared the decomposition rate of different species or functional groups of the same grassland community (Li et al., 2011; Shaw & Harte, 2001), measured the decay rate of above‐ versus belowground litter (Wang et al., 2015), or addressed both comparisons (Semmartin et al., 2004; Vivanco & Austin, 2006). Collectively, these studies showed that high N content of the litter generally increased, while high lignin content and lignin:N ratio decreased the decomposition rate. Microbial decomposition of lignin is usually the slowest among the three main fibrous components of plant biomass (lignin, cellulose, and hemicellulose; Shipley & Tardif, 2021; Yan et al., 2019), because only few organisms (in grasslands particularly white‐rot basidiomycete fungi; Deacon et al., 2006; Kabuyah et al., 2012) can produce the enzymes necessary for lignin degradation. These previous field experiments also showed that the initial decomposition rate (within the first year) was higher for hemicellulose than for cellulose (Shipley & Tardif, 2021; Yan et al., 2019). Thus, in our study, the higher cellulose content and the lower hemicellulose content of roots compared to leaves may have contributed to the lower decomposition rate of roots. In addition, the faster decomposition of leaves compared to roots may also be related to the food preference of Collembola. In a laboratory experiment, each of the three Collembola species studied (including *Folsomia candida*, which occurs at our study site; Flórián et al., 2019) preferred leaves to roots of both *F. vaginata* and *S. borysthenica* (Seres et al., 2018).

### 4.3 Effect of soil depth

The greater mass loss of both leaf and root litter in the 10–15 cm layer compared to the 0–5 cm layer of the soil is in line with our hypothesis (H_4_) that the rate of decomposition is faster in the deep soil than in the shallow soil. This result is most likely attributed to the more favorable temperature and moisture conditions for decomposition in the subsoil compared to the topsoil, where extreme edaphic conditions may limit the activity of decomposers. In sandy soil, which has low water‐holding capacity (Kovács‐Láng et al., 2000), the uppermost soil layer (0–5 cm) can completely dry out in summer, while at the same time, soil water content gradually increases with depth in the main rooting zone of the dominant grasses (5–40 cm; Kovács‐Láng, Lhotsky, et al., 2006). In addition, the topmost soil layer can strongly warm up in summer (e.g. Hargitai (1940) measured 67°C on the soil surface, but 28–29°C at 20 cm depth in the soil of a *F. vaginata* grassland in July). Similar to our results, faster decomposition was observed in the deeper soil layer than in the upper layer of the soil in grasslands (Castanha et al., 2018; Garcia‐Pausas et al., 2012) and in a Mediterranean experimental field (Rovira & Vallejo, 1997) due to more suitable microclimatic conditions for litter decay in the deeper soil layer. By contrast, some other studies in grasslands reported slower decomposition in the deeper layer than in the shallow layer of the soil, which may be due to a different vertical distribution (i.e., gradual decline) of soil moisture content along the studied soil profile (Gill et al., 1999; Gill & Burke, 2002), or to the fact that factors other than soil microclimate play the dominant role in controlling litter decomposition (Von Haden & Dornbush, 2019).

### 4.4 Implications for drought‐induced dominance shift

Our results on different decomposition rate between the two grass species, plant parts, and soil depths, and that these differences were similar in magnitude to the direct effect of drought on decomposition, suggest that changes in litter quality, root‐to‐shoot ratio, and rooting depth should also be studied to assess the overall effect of drought on decomposition. Each of these changes may be related to altered biomass or nutrient allocation within a species in response to drought, and/or a drought‐induced change in species dominance. Within species, plants that experience water stress often have greater root‐to‐shoot ratios or deeper roots than well‐watered plants (Fernández & Reynolds, 2000; Huang, 1999; Skinner & Comas, 2010; Wang et al., 2016). In semi‐arid environments, drought also tends to increase the C:N ratio in leaves, thus decreasing litter quality (Sardans et al., 2012). In our experiment, rain exclusion clearly resulted in a shift in dominance toward *S. borysthenica* at the expense of *F. vaginata* (see Table 1 and the ‘Section 2.3’), similar to which was observed in a previous study following extreme natural droughts (in 2000 and 2003) in the study area (Kovács‐Láng, Kröel‐Dulay, et al., 2006). Our results showed that *S. borysthenica* generally had a lower decomposition rate most likely due to its poorer litter quality (lower N content and higher lignin content) compared to *F. vaginata*. Therefore, an increased abundance of *S. borysthenica* with a simultaneous decrease in the cover of *F. vaginata* in response to drought may amplify the direct negative effect of drought on decomposition indirectly through the impact of litter quality. At the same time, an earlier study reported a lower root‐to‐shoot ratio for *S. borysthenica* than for *F. vaginata* at the study site (Lhotsky et al., 1998), thus a shift in dominance from *F. vaginata* to *S. borysthenica* may increase the ratio of shoot to root biomass at the level of dominant species. As we found faster decomposition for leaves than for roots, this shift in biomass allocation may partly offset the negative effects of drought on decomposition. The negative effects may also be alleviated by the deeper roots of *S. borysthenica* (10–40 cm) compared to that of *F. vaginata* (5–30 cm; Simon & Batanouny, 1971; Kalapos, 1994), because in our study, the mass loss of roots was greater in the deep soil layer compared to the shallow layer. Thus, our results suggest that a shift in species abundance in response to drought may result in multiple indirect effects on decomposition, such as those arising from changes in litter quality, biomass allocation, and rooting depth. These indirect effects may be in complex interactions with each other and with the direct drought effect, which highlights the need to measure the decomposition rate of community‐specific litter for a better understanding of the effects of drought on litter decomposition. In addition, further studies are required to evaluate whether a single‐year extreme drought has medium‐ or long‐term legacy effects on litter decomposition in the post‐drought years.

Numerous studies in grasslands documented shifts in plant species abundances in response to natural or experimental drought (Albertson & Weaver, 1942; Breman & Cissé, 1977; Gibbens & Beck, 1987; Hoover et al., 2014; Jin et al., 2019), either by reordering of species or functional groups (i.e., some species become abundant under changing conditions at the expense of others; Smith et al., 2009), or through the replacement of a dominant species. When a shift in species abundances occurs, the litter decomposition rate of species that are favored under altered environmental conditions may differ from that of previously abundant species. In grasslands, such differences in the decomposition rate of species that shift in dominance were reported in response to experimental warming (Harte & Shaw, 1995; Shaw & Harte, 2001), nitrogen addition (Li et al., 2011; Zeng et al., 2010), or grazing (Semmartin et al., 2004). Some previous studies in shrublands and forests showed that drought may also induce such a difference. For example, in a desert shrubland, the abundance of the easily decomposable *Lycium pallidum* decreased in a dry year compared to a wet year to the advantage of *Larrea tridentata*, which had a lower decomposition rate (Weatherly et al., 2003). In a mixed Mediterranean forest where *Quercus ilex* gradually replaced the previously dominant *Pinus sylvestris* in response to drought, Barba et al. (2016) found a faster decomposition of oak leaves compared to pine needles, while a slower decomposition of oak roots compared to pine roots. However, to our knowledge, our study is the first to demonstrate different decomposition rates of dominant species that shift in dominance in response to drought in grasslands.

## 5 CONCLUSIONS

Our field experiment revealed that a 5‐month extreme drought substantially decreased the decomposition of both leaf and root litter of two dominant perennial grass species in a semi‐arid temperate grassland, most likely due to the reduction of the biological activity of decomposer organisms. In addition, we found differences in decomposition rate between the two species, plant parts, and soil depths in a magnitude similar to the direct drought effect. These differences indicate that droughts and drier climate may also alter decomposition indirectly through changes in litter quality, root‐to‐shoot ratio, and rooting depth; and these indirect effects may amplify or attenuate the direct impact of drought. Our study also emphasizes the importance of shifts in plant species abundances in response to drought, which can mediate these indirect effects of drought on decomposition. Since drought induces many parallel changes in ecosystems, and our results highlight that these changes may in turn modify decomposition, we conclude that for a reliable estimate of decomposition rates under climate change, both direct and indirect climatic effects, as well as their complex interactions should be considered.

## AUTHOR CONTRIBUTIONS

**Anikó Seres:** Conceptualization (equal); data curation (equal); formal analysis (equal); investigation (equal); methodology (equal); project administration (equal); validation (equal); visualization (equal); writing – original draft (equal); writing – review and editing (equal). **György Kröel‐Dulay:** Conceptualization (equal); funding acquisition (lead); investigation (equal); methodology (equal); project administration (equal); supervision (equal); validation (equal); writing – review and editing (lead). **Judit Szakálas:** Investigation (equal); validation (equal); writing – review and editing (equal). **Péter István Nagy:** Conceptualization (equal); investigation (equal); methodology (equal); project administration (equal); validation (equal); writing – review and editing (equal). **Gergely Boros:** Investigation (equal); validation (equal); writing – review and editing (equal). **Gábor Ónodi:** Data curation (equal); funding acquisition (equal); investigation (equal); validation (equal); writing – review and editing (equal). **Miklós Kertész:** Investigation (equal); validation (equal); writing – review and editing (equal). **Katalin Szitár:** Formal analysis (lead); writing – review and editing (equal). **Andrea Mojzes:** Data curation (equal); visualization (equal); writing – original draft (equal); writing – review and editing (lead).

## CONFLICT OF INTEREST

The authors declare that they have no conflict of interest.

## Supporting information


Figure S1‐S2
Table S1‐S3

## Data Availability

The data that support the findings of this study are available in Figshare (Seres et al., 2022).
