# Supplementary material for: The response of litter decomposition to extreme drought modified by plant species, plant part, and soil depth in a temperate grassland
Source: Ecol Evol. 2022 Dec 21;12(12):e9652. doi: 10.1002/ece3.9652 (PMC9772495; doi:10.1002/ece3.9652)
Supplement: Supplementary file 1 — Figure S1‐S2 Table S1‐S3 [file ECE3-12-e9652-s001.pdf]

Supporting Information

The response of litter decomposition to extreme drought modified by plant species, plant part, and soil depth in a temperate grassland

Anikó Seres[1], György Kröel-Dulay[2], Judit Szakálas[1], Péter István Nagy[1], Gergely Boros[1,2], Gábor Ónodi[2], Miklós Kertész[2], Katalin Szitár[3], Andrea Mojzes[2]

[1] Department of Zoology and Ecology, Institute for Wildlife Management and Nature Conservation, Szent István Campus, Hungarian University of Agriculture and Life Sciences, Gödöllő, Hungary

[2] Institute of Ecology and Botany, Centre for Ecological Research, Vácrátót, Hungary

[3] 'Lendület' Landscape and Conservation Ecology Group, Institute of Ecology and Botany, Centre for Ecological Research, Vácrátót, Hungary

Contact Information

Corresponding author: Andrea Mojzes
Address: Institute of Ecology and Botany, Centre for Ecological Research, Alkotmány u. 2-4, H-2163, Vácrátót, Hungary
E-mail: mojzes.andrea@ecolres.hu
Tel.: +36 28 360122; +36 28 360147
Fax: +36 28 360110
ORCID ID: 0000-0003-2171-403X

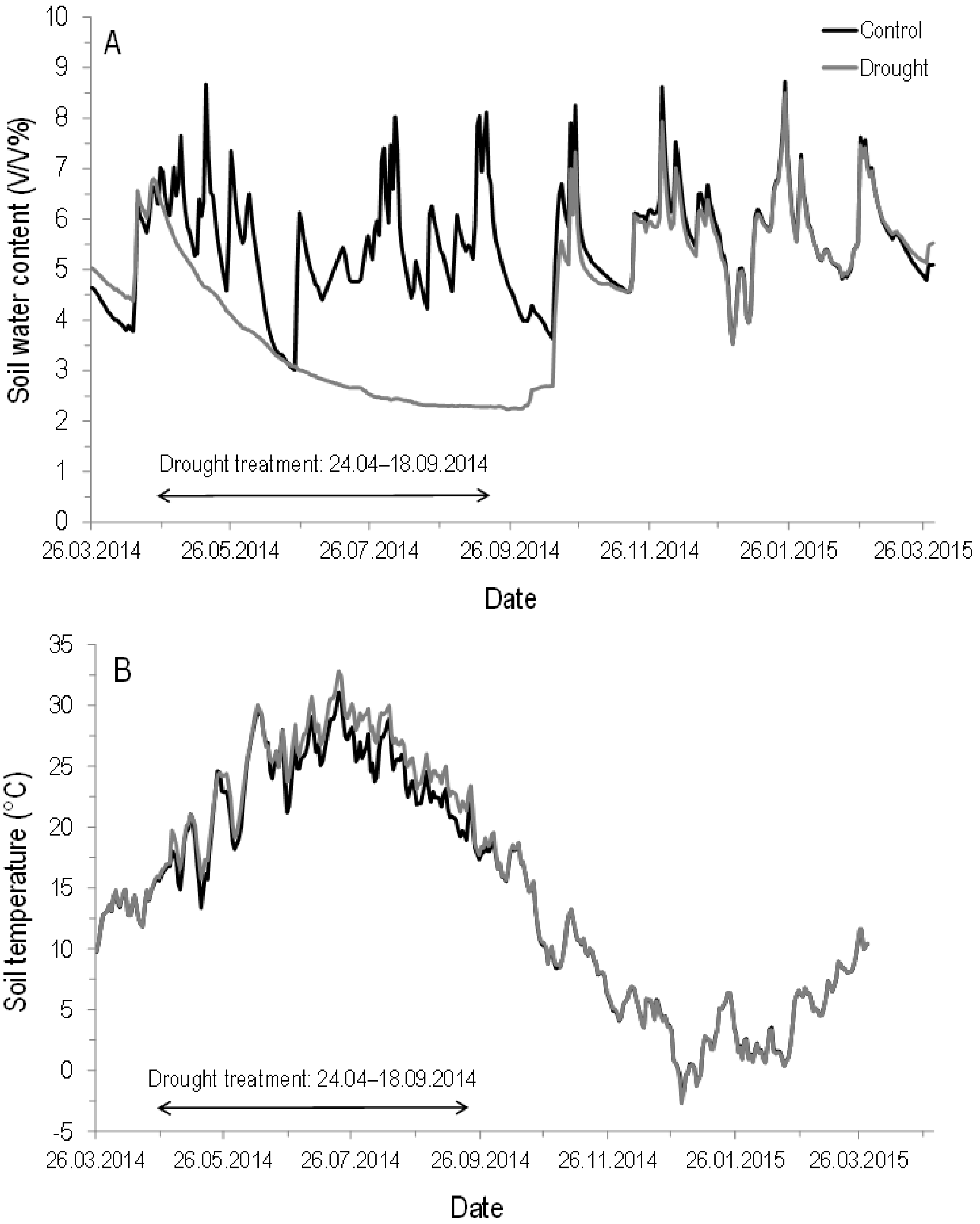

Fig. S1 (**A**) Mean volumetric soil water content at 0–30 cm depth and (**B**) mean soil temperature at 10 cm depth in the control and drought plots during the experiment. Arrows indicate the timing of the drought treatment.

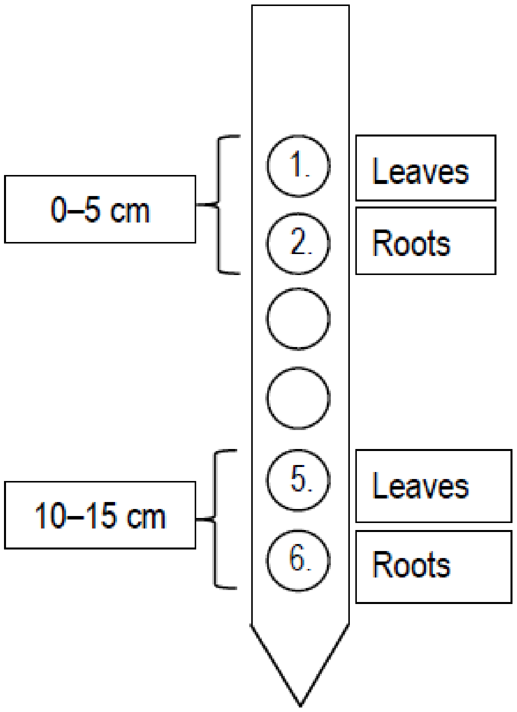

Fig. S2 Design of the minicontainer system used in our experiment. Four minicontainers (MCs) were placed into the two upper and the two bottom holes of a six-hole PVC bar. The third and fourth holes were left empty. The first and fifth MCs were filled with leaves, and the second and sixth MCs were filled with roots. The bar was inserted vertically into the soil (the two upper MCs at the 0–5 cm layer, and the two bottom MCs at the 10–15 cm layer).

Table S1

Results of linear mixed-effects (LME) models for the whole dataset of the percentage mass loss of leaf and root litter with explanatory variables, i.e. treatment (control vs. drought), species (*Festuca vaginata* or *Stipa borysthenica*), soil depth (shallow vs. deep soil layer), and time (2, 4, 6, or 12 months), and their interactions. (**A**) Minimum adequate models, where non-significant interaction terms were removed during the model selection. (**B**) Full models with all interaction terms. Data for leaves and roots were analysed separately. df, χ^2, and *P* values denote the degrees of freedom, test statistics, and significance levels, respectively. Bold *P* values are significant at 0.05.

**A**	Leaves			Roots		
Source of variation	df	χ^2	*P*	df	χ^2	*P*
Treatment	1	110.24	< **0.001**	1	52.18	< **0.001**
Species	1	72.52	< **0.001**	1	40.04	< **0.001**
Depth	1	222.61	< **0.001**	1	17.44	< **0.001**
Time	3	193.67	< **0.001**	3	180.43	< **0.001**
Treatment × Species	1	0.28	0.596			
Treatment × Depth	1	0.03	0.862			
Treatment × Time	3	37.10	< **0.001**	3	21.99	< **0.001**
Species × Time	3	2.28	0.515			
Depth × Time	3	16.93	< **0.001**			
Treatment × Species × Time	3	16.58	< **0.001**			
Treatment × Depth × Time	3	9.54	**0.023**			

**B**	Leaves			Roots		
Source of variation	df	χ^2	*P*	df	χ^2	*P*
Treatment	1	110.47	< **0.001**	1	52.60	< **0.001**
Species	1	73.12	< **0.001**	1	40.37	< **0.001**
Depth	1	223.00	< **0.001**	1	17.59	< **0.001**
Time	3	195.81	< **0.001**	3	181.90	< **0.001**
Treatment × Species	1	0.32	0.571	1	2.20	0.138
Treatment × Depth	1	0.02	0.879	1	1.52	0.218
Species × Depth	1	0.21	0.645	1	0.93	0.334
Treatment × Time	3	37.70	< **0.001**	3	22.16	< **0.001**
Species × Time	3	2.32	0.509	3	3.34	0.343
Depth × Time	3	17.15	< **0.001**	3	4.08	0.253
Treatment × Species × Depth	1	2.07	0.150	1	1.68	0.195
Treatment × Species × Time	3	16.78	< **0.001**	3	1.17	0.761
Treatment × Depth × Time	3	9.69	**0.021**	3	3.87	0.276
Species × Depth × Time	3	3.44	0.328	3	2.18	0.537
Treatment × Species × Depth × Time	3	3.37	0.338	3	2.51	0.473

Table S2

Results of linear models (LMs) for the 12-month data of the percentage mass loss of leaf and root litter with explanatory variables, i.e. treatment (control vs. drought), species (*Festuca vaginata* or *Stipa borysthenica*), and soil depth (shallow vs. deep soil layer), and their interactions (full models with all interaction terms). Data for leaves and roots were analysed separately. df, F, and *P* values denote the degrees of freedom, test statistics, and significance levels, respectively. Bold *P* values are significant at 0.05.

	Leaves			Roots		
Source of variation	df	F	*P*	df	F	*P*
Treatment	1	90.44	**< 0.001**	1	28.09	**< 0.001**
Species	1	21.92	**< 0.001**	1	5.63	**0.023**
Depth	1	102.72	**< 0.001**	1	9.11	**0.004**
Treatment × Species	1	5.04	**0.030**	1	1.51	0.226
Treatment × Depth	1	3.32	0.076	1	2.14	0.152
Species × Depth	1	0.00	0.946	1	0.01	0.916
Treatment × Species × Depth	1	2.08	0.157	1	1.72	0.197

Table S3

Results of generalised least squares (GLS) models for initial litter quality (chemical composition) including carbon (C) and nitrogen (N) contents, C:N ratio, and the contents of lignin, cellulose, and hemicellulose with explanatory variables, i.e. species (*Festuca vaginata* or *Stipa borysthenica*) and plant part (leaf vs. root), and their interaction (full models with all interaction terms). df, χ^2, and P values denote the degrees of freedom, test statistics, and significance levels, respectively. Bold P values are significant at 0.05.

Source of variation	df	χ^2	P
Carbon (C)			
Species	1	6.96	**0.008**
Plant part	1	39.56	< **0.001**
Species × Plant part	1	36.26	< **0.001**
Nitrogen (N)			
Species	1	281.17	< **0.001**
Plant part	1	535.65	< **0.001**
Species × Plant part	1	164.23	< **0.001**
C:N ratio			
Species	1	291.07	< **0.001**
Plant part	1	354.23	< **0.001**
Species × Plant part	1	163.25	< **0.001**
Lignin			
Species	1	4135.35	< **0.001**
Plant part	1	15359.52	< **0.001**
Species × Plant part	1	0.46	0.500
Cellulose			
Species	1	1.21	0.272
Plant part	1	1595.00	< **0.001**
Species × Plant part	1	463.45	< **0.001**
Hemicellulose			
Species	1	405.60	< **0.001**
Plant part	1	388.68	< **0.001**
Species × Plant part	1	219.84	< **0.001**
